# Thermally Rearranged (TR) Polybenzoxazoles from *o*-Substituted Precursor Polyimides with Phenyl Pendant Groups: Synthesis, Properties, and Thermal Rearrangement Conditions

**DOI:** 10.1021/acs.macromol.4c00169

**Published:** 2024-08-06

**Authors:** Mario Rojas-Rodriguez, Sandra Rico-Martínez, Pedro Prádanos, Cristina Álvarez, Larissa Alexandrova, Young Moo Lee, Ángel E. Lozano, Carla Aguilar-Lugo

**Affiliations:** 1 Instituto de Investigaciones en Materiales, Universidad Nacional Autónoma de Mexico, Circuito Exterior S/N, Ciudad Universitaria, 04510 Ciudad de Mexico, Mexico; 2 Instituto Universitario CINQUIMA, University of Valladolid, Paseo Belén 5, 47011 Valladolid, Spain; 3 SMAP, Associated Research Unit to CSIC, Faculty of Science, University of Valladolid, Paseo Belén 7, 47011 Valladolid, Spain; 4 Instituto de Ciencia y Tecnología de Polímeros, ICTP-CSIC, Juan de la Cierva 3, E-28006 Madrid, Spain; 5 Department of Energy Engineering, College of Engineering, Hanyang University, Seoul 04763, Republic of Korea

## Abstract

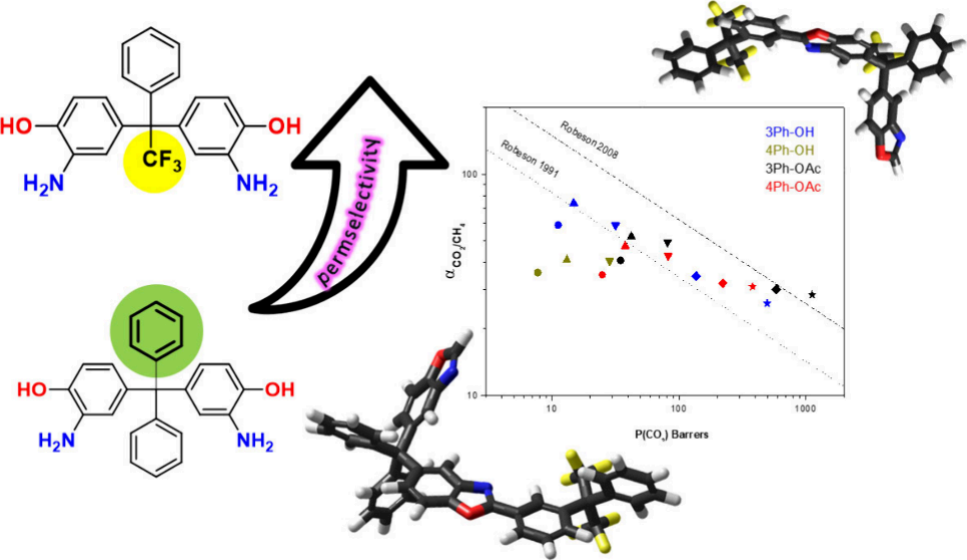

A series of polyimides (PIs) was synthesized from 6FDA and two *o*-OH substituted diamines having bulky pendant phenyl, Ph, and trifluoromethyl, CF_3_, groups as precursors for thermally rearranged polybenzoxazole, TR-PBO, membranes. One diamine had two pendant Ph substituents; in the other, the substituents were Ph and CF_3_. Applying azeotropic and chemical cyclizations allowed the obtention of four *o*-hydroxy (*o*-OH) or/and *o*-acetoxy (*o*-OAc) substituted PIs depending on the imidization method. The PIs were labeled as 3Ph-OH, 4Ph-OH, or 3Ph-OAc and 4PH-OAc, respectively. Thermal rearrangements of all four precursors were investigated in the interval from 350 to 450 °C. The conversions to TR-PBO increased with temperature, and almost quantitative conversions were obtained at temperatures close to 450 °C, although *o*-OH substituted PIs reached conversions slightly higher than those of *o*-OAc PIs at a given temperature. The TR-polymers’ fractional free volume (FFV) also increased with conversion but was higher for the *o*-OAc substituted precursors. Despite the high TR-PBO conversions, self-supported uniform TR membranes with reasonable mechanical properties were obtained, except for 4Ph-OH. Gas separation behavior of the membranes significantly improved after the thermal treatment, and the final CO_2_/CH_4_ permselectivities lay between the 1991 and 2008 Robeson upper bounds. Particularly, TR-membranes derived from *o*-OAc precursors and with pendant CF_3_ group demonstrated better gas transport properties with values of *P*_(CO2)_ = 1121 barrer and α_CO2/CH4_ = 29 for 3Ph-OAc derived membrane, which positioned it beyond the 2008 upper limit.

## Introduction

Gas separation using polymeric membranes is an intensively developing technique with multiple applications: nitrogen separation from air, CO_2_ removal from natural gas, hydrogen from condensable hydrocarbons, and other emerging applications such as helium recovery and biogas treatment.^1−4^ Polymeric membranes offer various advantages compared to traditional methods: low-cost manufacturing, easy handling, processability, reduced environmental impact, and simplification of operation.^5,6^ However, the full industrial implementation of conventional polymeric membranes has been limited primarily because of an intrinsic trade-off behavior between permeability and selectivity^7,8^ and the inability to maintain long-term gas separation performance due to physical aging and plasticization.^9−11^ Many efforts have been made to overcome these limitations; the first breakthrough was achieved with the development of polymers of intrinsic microporosity (PIMs).^12,13^ PIMs have stiff, contorted macromolecular chains, which cannot pack efficiently, generating enhanced porosity. Due to these specific structural features, PIMs demonstrate extraordinary performance as membrane materials, with permeability similar to ultraporous polymers like polytrimethylsilylpropyne, PTMSP, but with much higher selectivity.^14,15^ Although intense research in this area has allowed the obtention of longer-lasting materials with permselectivity much above the Robeson limit,^16−18^ the elaborated and costly synthesis of PIMs has hindered their industrial application.^19^ Another milestone in polymer membranes is related to the development of thermally rearranged polymers (TR polymers). These materials are obtained by high-temperature treatment of polymeric precursors in the solid state, which are mostly functionalized aromatic polyimides bearing OH substituents in the *ortho* position to the imidic nitrogen.^20−22^ A heat treatment of the *o*-hydroxypolyimides (*o*-HPI) produces thermally rearranged polybenzoxazoles (TR-PBOs).^23−26^ During the TR process, the membrane’s fractional free volume (FFV) distribution changes, forming bottleneck-type structures^27−30^ that produce materials with outstanding permeabilities.^16−18^ Thanks to their rigid rod-like structure with high-torsional energy barriers, these TR-PBOs demonstrate a low tendency to plasticization and suffer less from physical aging.^31,32^ Furthermore, PBOs are distinguished from most high performance polymers by their higher thermal and chemical stabilities, and therefore, they are excellent candidates for applications under severe conditions such as hydrogen purification.^33,34^ However, for further development of TR materials, several problems related to their practical implementation have to be resolved, and among them, an improvement of the mechanical properties is particularly important. Generally, conversions of *o*-HPIs to TR-PBOs occur in a temperature range of 300–450 °C, which is close enough to the initial degradation, and therefore, this thermal treatment deteriorates the mechanical properties to some extent.^35−37^ Thus, the search for novel structures to obtain precursors capable of more efficient conversion at lower temperatures is necessary. Here we report two new monomers with phenyl pendant groups, 1,1-bis(3-amino-4-hydroxyphenyl)-1-phenyl-2,2,2-trifluoroethane (**AH3P**) and bis(3-amino-4-hydroxyphenyl) biphenyl methane (**AH4P**) designed for the synthesis of novel *o*-HPIs. Their conversion to TR-BPOs was studied, and the gas transport properties of the membranes before and after thermal treatment are discussed.

## Experimental Section

### Materials

Concentrated nitric acid (65%), glacial acetic acid, toluene, dichloromethane, chloroform, and tetrahydrofuran (THF) were reagent grade from Baker and used as received. Phenol (99%), 2,2,2-trifluoroacetophenone (99%), methanesulfonic acid (MSA) (99%), 3-mercaptopropionic acid (99%), trifluoromethanesulfonic acid (TFSA) (98%), hydrazine monohydrate (98%), palladium 10 wt % on activated carbon, chlorotrimethyl silane (CTMS), pyridine (Py), *N*,*N*-dimethylaminopyridine (DMAP), *N*-methyl-2-pyrrolidinone (NMP), *N*,*N*-dimethylacetamide (DMAc), and *o*-xylene were all purchased from Aldrich and used without any additional purification. 4,4′-Dihydroxytetraphenylmethane was purchased from TCI Europe and used as received. 2,2′-Bis(3,4-dicarboxyphenyl)hexafluoropropane dianhydride (6FDA) was provided by Cymit Qumica and was sublimed at 220 °C prior to use.

### Synthesis of 1,1-Bis(4-hydroxyphenyl)-1-phenyl-2,2,2-trifluoroethane (**1**)

In a three-necked flask, 5.7 g (60.0 mmol) of molten phenol was dissolved in 15 mL of toluene at 50 °C under a nitrogen atmosphere, and then 4.0 mL (28.5 mmol) of 2,2,2-trifluoroacetophenone was added, and the mixture was left stirring until its complete homogenization (5–10 min). Then, 0.2 mL of 3-mercaptopropionic acid, 2.0 mL of MSA, and 0.2 mL of TFSA were added dropwise. The reaction was carried out for 12 h at 50 °C. Thenceforth, the reaction was cooled to room temperature, and a white precipitate formed was filtered off, washed with hot water, and recrystallized from ethanol giving colorless needles. 75% Yield. ^1^H NMR (400 MHz, DMSO-*d*_6_) δ 9.61 (s, 2H), 7.39–7.34 (m, 3H), 7.06 (d, *J* = 6.6 Hz, 2H), 6.83 (d, *J* = 8.7 Hz, 4H), 6.75 (d, *J* = 8.7 Hz, 4H). ^13^C NMR (101 MHz, DMSO-*d*_6_) δ: 157.09, 140.78, 130.88, 130.31, 129.62, 128.56, 127.99, 115.30, 63.61.

### Synthesis of 1,1-Bis(3-nitro-4-hydroxyphenyl)-1-phenyl- 2,2,2-trifluoroethane (**2**)

8.0 g (23.2 mmol) of **1** was dispersed in 60 mL of a mixture of toluene with glacial acetic acid (50/50 v/v) at room temperature, and the dispersion was cooled to 0 °C. Afterward, 7.5 mL of concentrated nitric acid was added dropwise over 1 h. After stirring for an additional hour at 0 °C, the temperature was left to reach room temperature and the reaction was maintained for 2 h at this temperature. Then, the reaction solution was poured into cold water and the dark yellow precipitate was separated by filtration, washed with water, and then purified by silica gel column chromatography with dichloromethane as an eluent. Yellow product was obtained with 72% yield. ^1^H NMR (400 MHz, DMSO-*d*_6_) δ 11.46 (s, 2H), 7.51–7.48 (m, 5H), 7.37 (dd, *J* = 8.9, 2.0 Hz, 2H), 7.27 (d, *J* = 8.9 Hz, 2H), 7.16 (d, *J* = 4.2 Hz, 2H). ^13^C NMR (101 MHz, DMSO-*d*_6_) δ: 152.59, 138.43, 136.67, 136.25, 129.74, 129.62, 129.41, 129.07, 126.42, 120.26, 63.49.

### Synthesis of 1,1-Bis(3-amino-4-hydroxyphenyl)-1-phenyl-2,2,2-trifluoroethane (**AH3P**)

A mixture of 10.0 g (23.0 mmol) of **2**, 250 mL of ethanol, and 1.0 g of 10% palladium on carbon (Pd/C) was heated at 75 °C under nitrogen followed by the slow addition of 20 mL of hydrazine monohydrate. After that, the solution was heated and maintained at reflux for 24 h. The reaction mixture was filtered through Celite to remove the catalyst, concentrated in a rotary evaporator, and finally poured into cold water. The resulting white solid was filtered off, washed with water, and recrystallized from a mixture of ethanol/water (50/50 v/v). 85% yield. ^1^H NMR (400 MHz, DMSO-*d*_6_) δ 9.13 (s, 2H), 7.35–7.28 (m, 3H), 7.08 (d, *J* = 7.0 Hz, 2H), 6.56 (d, *J* = 8.3 Hz, 2H), 6.45 (s, 2H), 5.98 (dd, *J* = 8.3, 1.8 Hz, 2H), 4.52 (s, 4H). ^13^C NMR (101 MHz, DMSO-*d*_6_) δ: 143.78, 141.52, 136.40, 131.58, 129.97, 128.22, 127.70, 118.61, 116.17, 113.82, 64.14.

### Synthesis of Bis(3-nitro-4-hydroxyphenyl) Biphenyl Methane (**3**)

Concentrated nitric acid (11.0 mL) was dropped into a stirred solution of 12.0 g (34.0 mmol) of 4,4′-dihydroxytetraphenylmethane in 90 mL of toluene/glacial acetic acid (50/50 v/v) mixture at 0 °C. The mixture was stirred for 2 h at 0 °C and then removed from the ice to react for another 2 h at room temperature. The solid product was filtered off and washed with cold water and then methanol. The filtered solid was purified by silica gel column chromatography with dichloromethane as an eluent to obtain a yellow product with a 68% yield. ^1^H NMR (400 MHz, DMSO-*d*_6_) δ 11.09 (s, 2H), 7.65 (d, *J* = 2.4 Hz, 2H), 7.36 (t, *J* = 7.4 Hz, 4H), 7.28 (t, *J* = 7.4 Hz, 2H), 7.25 (dd, *J* = 8.9, 2.4 Hz, 2H), 7.14–7.10 (m, 6H). ^13^C NMR (101 MHz, DMSO-*d*_6_) δ: 148.31, 142.34, 138.46, 135.51, 131.03, 127.40, 125.64, 119.26, 118.12, 113.35, 63.77.

### Synthesis of Bis(3-amino-4-hydroxyphenyl) Biphenyl Methane (**AH4P**)

A mixture of 8.0 g (18.0 mmol) of **3**, 120 mL of ethanol, and 0.8 g of 10% palladium on carbon (Pd/C) was added to a three-neck flask under a nitrogen atmosphere. The solution was heated at 75 °C, and 18 mL of hydrazine monohydrate was slowly added, which was then maintained at reflux for 24 h. The reaction mixture was filtered using Celite to remove the Pd/C catalyst, concentrated in a rotary evaporator, and poured into water. The obtained white solid was filtered, washed with water, and recrystallized from ethanol to afford 80% Yield. ^1^H NMR (400 MHz, DMSO-*d*_6_) δ 8.86 (s, 1H), 7.26–7.10 (m, 10H), 6.51 (d, *J* = 8.2 Hz, 2H), 6.39 (d, *J* = 1.8 Hz, 2H), 6.18 (dd, *J* = 8.2, 1.8 Hz, 2H), 4.36 (s, 4H). ^13^C NMR (101 MHz, DMSO-*d*_6_) δ 148.31, 142.34, 138.46, 135.51, 131.03, 127.40, 125.64, 119.26, 118.12, 113.35, 63.77. ^13^C NMR (101 MHz, DMSO-*d*_6_) δ: 143.78, 141.52, 136.40, 131.58, 129.97, 128.22, 127.70, 118.61, 116.17, 113.82, 64.00, 40.61, 40.40, 40.19, 39.98, 39.77, 39.56, 39.35.

### Synthesis of Polyimides Derived from 6FDA and **AH3P** and **AH4P**

*Ortho*-hydroxy (*o*-OH PI) and *ortho*-acetoxy (*o*-OAc PI) polyimides were prepared using the base-assisted silylation method via a two-step procedure with hydroxy-poly(amic acid) (HPAA) intermediates using azeotropic and chemical imidization correspondingly. Depending on the type of imidization, a different solvent was used for the obtention of HPAA; in the case of chemical, DMAc was used, while NMP was used for the azeotropic one. HPAA prepolymers were synthesized using a three-necked flask with a mechanical stirrer and nitrogen atmosphere. The reaction system was charged with 5.0 mmol of diamine (**AH3P** or **AH4P**) and 5.0 mL of the corresponding solvent. Once the diamine was completely dissolved, the solution was cooled to 0 °C, and the corresponding amounts of CTMS (1 mol/mol reactive group), Py (1 mol/mol reactive group), and DMAP (0.1 mol/mol Py) were added. After adding silylation agents, the temperature was allowed to reach room temperature, and the mixture was stirred for 15 min to allow formation of the silylated diamine. The solution was cooled again to 0 °C, and 6FDA (5.0 mmol) and 5.0 mL of solvent were added. The reaction mixture was stirred for 15 min at this temperature, slowly raised to room temperature, and left overnight to obtain the HPAA.

To obtain the *o*-OAc PIs, a mixture of acetic anhydride (40 mmol, 4 mol/mol reactive group) and Py (40 mmol, 4 mol/mol reactive group) was added to the HPAA solution. The solution was stirred for 6 h at room temperature and for 1 h at 60 °C to ensure complete imidization. The final polyimide solution was cooled, poured into water, and washed with water/ethanol (50/50 v/v). The o-OAc PIs fibers were dried in a vacuum oven at 150 °C until a constant weight. The *o*-OH PI was prepared by adding 10 mL of *o*-xylene, as an azeotropic agent, to the HPAA solution. The reaction mixture was heated at 180 °C for 6 h to allow the cyclization. Finally, the *o*-xylene was removed using nitrogen flow, and the polyimide was poured into water, washed with water/ethanol (50/50 v/v), and dried under vacuum at 150 °C until constant weight.

### Film Casting and Thermal Treatment

10 wt % polyimides solutions were prepared in THF (*o*-OH PI) or chloroform (*o*-OAc PI) and filtered through a 3.1 μm glass-fiber syringe filter. The solutions were cast onto glass plates, and the solvent was removed by evaporation. The films were removed from the plates and washed with deionized water. All the films were dried in a vacuum oven following a temperature step protocol as follows: 4 h at 120 °C, 3 h at 180 °C, 1 h at 200 °C, and 20 min at 250 °C. Polyimide films were thermally treated to PBOs using a Carbolite tube furnace under a nitrogen atmosphere. The procedure was as follows: all polyimide films were heated at 5 °C/min to 300 °C and kept isothermally for 1 h to ensure solvent removal and complete imidization. After that, the samples were heated at 5 °C/min to the final rearrangement temperature of 350 °C, 400 °C, 425 °C, or 450 °C and thus held off for the desired time (1 h or 30 min) and, finally, cooled to room temperature. The thermally treated membranes were labeled TR-*X*, where *X* indicates the final temperature applied to those samples.

### Characterization

The precursors and TR-PBOs films were characterized using a PerkinElmer Fourier transform infrared spectrometer (FT-IR) with an ATR accessory at a 4000–650 cm^–1^ scan range. ^1^H and ^13^C NMR were obtained on a Bruker 400 at 400 and 101 MHz, respectively. Differential scanning calorimetric (DSC) analyses were performed on a TA Instruments DSC Q-2000 analyzer (TA Instruments, DE, USA) at a 20 °C/min heating rate under a N_2_ atmosphere. A TA Instruments Q-500 thermobalance (TA Instruments, DE, USA) was used combined with a mass spectrometer (MS) ThermoStar GSD 301T (Pfeiffer Vacuum GmbH, Germany) to obtain thermogravimetric analyses (TGA) at 10 °C/min from 50 to 800 °C. To establish the thermal conditions for the TR samples, isothermal thermogravimetric analyses were performed using the previous TGA equipment and the following protocol: Film samples were thermally treated at 300 °C (1 h), then heated to the selected rearrangement temperature (350 °C, 400 °C, 425 °C, or 450 °C) at a heating rate of 5 °C/min and held isothermally for 4 h. The inherent viscosity of polymers was measured at 30 °C using a Canon-Ubbelohde suspended level viscometer and polymer solutions in NMP with a 0.5 g/dL concentration. Wide-angle X-ray scattering (WAXS) of all the studied samples was obtained on a Bruker D8 Advance system fitted with a Goebel mirror and a PSD Vantec detector with a Cu Kα (wavelength λ = 1.542 Å) radiation source using a scan rate of 0.5 s per step. The average *d*-spacing was obtained from the Bragg’s equation:
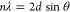
1where *n* is an integer number related to the Bragg order, *d* is the *d*-spacing, and θ is the scattering angle.

Density was measured with a XS105 dual range Mettler Toledo balance coupled with a density kit. The samples were weighed in air and into high-purity isooctane at room temperature, and the density was calculated from the following expression:
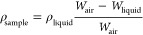
2

The fractional free volume (FFV) was calculated using the density data and the following equation:
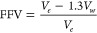
3where *V*_*e*_ is the polymer specific volume and *V*_*W*_ is the van der Waals volume. The van der Waals volume was calculated by molecular modeling using the Hyperchem computer program, version 8.0, and applying the semiempirical method Austin Model 1 (AM1).

A constant volume/variable pressure apparatus at 30 °C was used to evaluate the gas permeation properties for single gas feeds. The downstream pressure was kept below 10^–2^ mbar, while the upstream pressure was controlled at 3 bar for all gases. He, O_2_, N_2_, CH_4_, and CO_2_ gases were used for the measurements. The gas purities were up to 99.99% for all gases except CH_4_ and O_2_, which were 99.95%. Helium permeation tests were carried out at three upstream pressures (1, 3, and 5 bar) to confirm the lack of pinholes. Permeability values for the polyimides and their TR-PBOs films, *P*, were determined from the slope of downstream pressure vs time plotted (d*p*(*t*)/d*t*) in the steady state, according to the expression
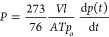
4where *V* (cm^3^), *l* (cm), and *A* (cm^2^), are respectively the downstream volume and thickness of the film and the effective area. *T* is the temperature in K, and *p*_*o*_ (cmHg) is the pressure of the feed gas in the upstream chamber. *P* is expressed in barrer [1 barrer = 10^–10^ (cm^3^ (STP) cm)/(cm^2^ scmHg). The ideal selectivity for a gas pair was calculated from the following relation:
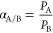
5where *P*_A_ and *P*_B_ are the permeability coefficients of pure gases A and B, respectively.

## Results and Discussion

### Monomer Synthesis

**Figure 1:**
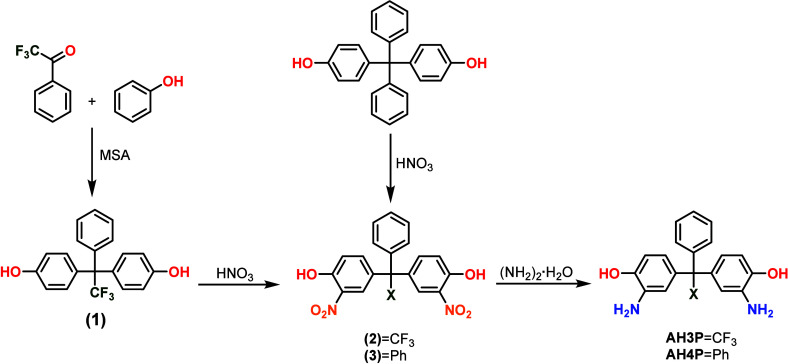
Monomer synthesis.

**Figure 2:**
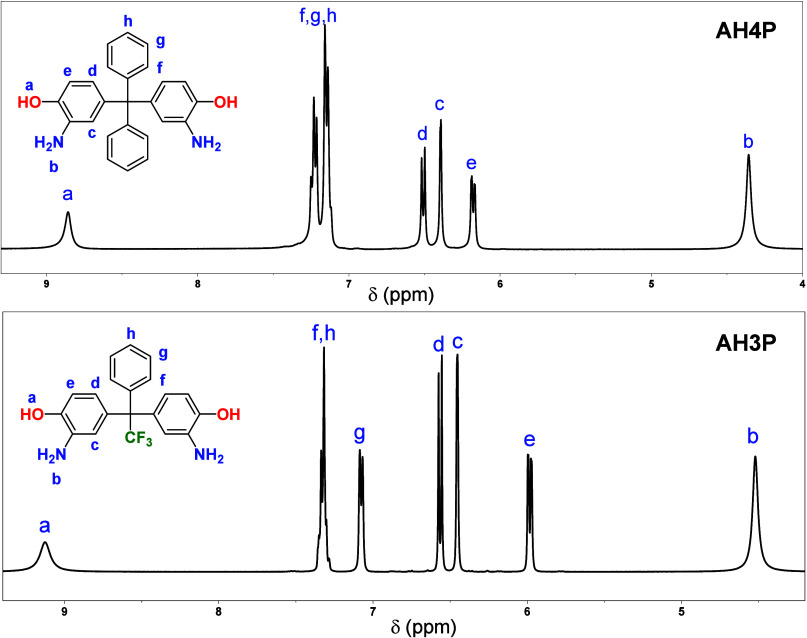
^1^H NMR spectra of the **AH3P** and **AH4P** monomers (400 MHz, DMSO-*d*_6_).

Diamine monomer, 1,1-bis(3-amino-4-hydroxyphenyl)-1-phenyl-2,2,2-trifluoroethane (**AH3P**), was synthesized from commercially available and inexpensive reagents by using an efficient three-step method (Figure 1). Bisphenol (**1**), 1,1-bis(4-hydroxyphenyl)-1-phenyl-2,2,2-trifluoroethane, was prepared from trifluoroacetophenone and phenol in acid media as described in the Experimental Section. The use of strong acids, such as MSA, gives better yields for the aromatic systems.^38^ Both dinitro compounds (**2, 3**) were obtained by direct nitration with a mixture of nitric acid/glacial acetic acid in good yields and with high purities. Finally, high purity monomers (**AH3P** and **AH4P**) in yields over 80% were obtained by reduction of nitro derivatives using hydrazine monohydrate and 10% Pd/C catalyst. The structures of the diamines and the intermediates were confirmed by ^1^H (Figure 2) and ^13^C NMR (Figures S1 and S2) spectroscopies. Both monomers exhibit the characteristic broad singlet corresponding to the OH protons (9.13 ppm for AH3P and 8.86 ppm for AH4P) and the singlets around 4.0–4.5 ppm consistent with NH_2_ protons.

In order to choose the best polymerization methodology, we studied the chemical reactivities of the monomers were studied. The reactivity of aromatic diamines depends to a great extent on their substituents; diamines bearing electron-withdrawing groups are less nucleophilic and exhibit lower reactivity. Table 1 shows the calculated HOMO values for **AH3P** and **AH4P** together with two other structurally similar diamines, which were named **APAF** and **APA**. **APAF** is a highly fluorinated diamine, 2,2-bis(3-amino-4-hydroxyphenyl) hexafluoropropane, with two CF_3_ groups, and **APA** is 2,2-bis(3-amino-4-hydroxylphenyl)propane, which has two methyl groups instead of CF_3_. The calculations were performed with the DFT method using the B3LYP/6-31G(d,p) basis set. **APAF** and **APA** were applied in polycondensation reactions, and polymers of reasonable molecular weights were obtained.^34,39−41^ The HOMO values correlate with electron-donating properties of the diamines and therefore reflect their reactivities. **APAF** has the lowest HOMO energy, which means that it is the least nucleophilic and reactive monomer among the four diamines. This should be expected because of the influence of the two electron-withdrawing CF_3_ groups in **APAF**. Contrarily, **APA** diamine presents the highest HOMO energy, which indicates the highest reactivity, thanks to the presence of two weakly electro-donating CH_3_ groups. The HOMO values for two synthesized diamines lie between these two extremes, with **AH3P** having a lower reactivity than **AH4P** due to the presence of CF_3_ groups. Thus, according to the simulations, **AH3P** and **AH4P** monomers should produce high molecular weight polymers. However, to ensure enough molecular weight to obtain films with good mechanical properties, a silylation methodology was used to increase the reactivity of the diamines.^34,42,43^

**Table 1:**
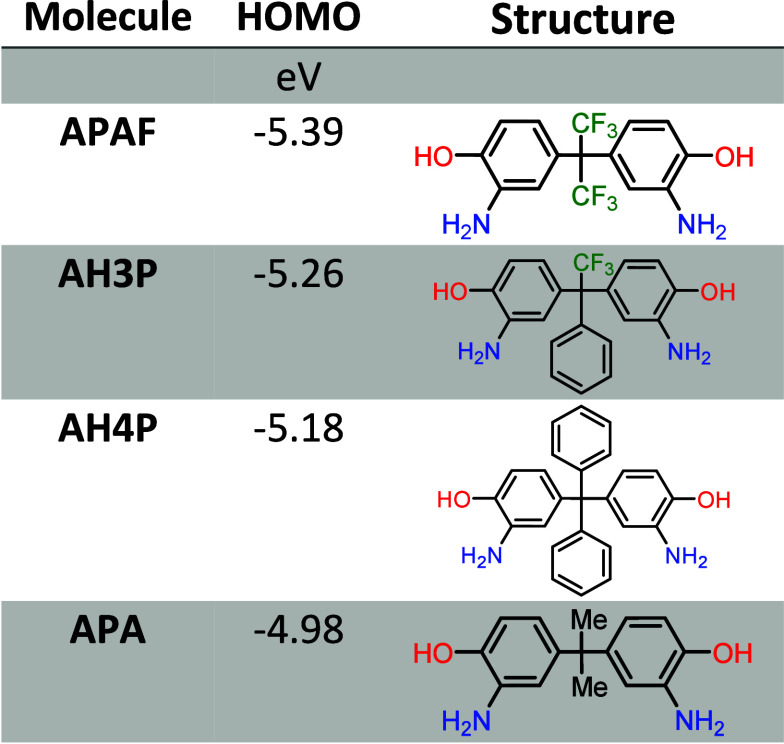
Calculated DFT-B3LYP 6-31G** HOMO Energies of Aromatic *o*-Hydroxydiamines

### Synthesis and Characterization of the Precursor Polyimides

**Figure 3:**
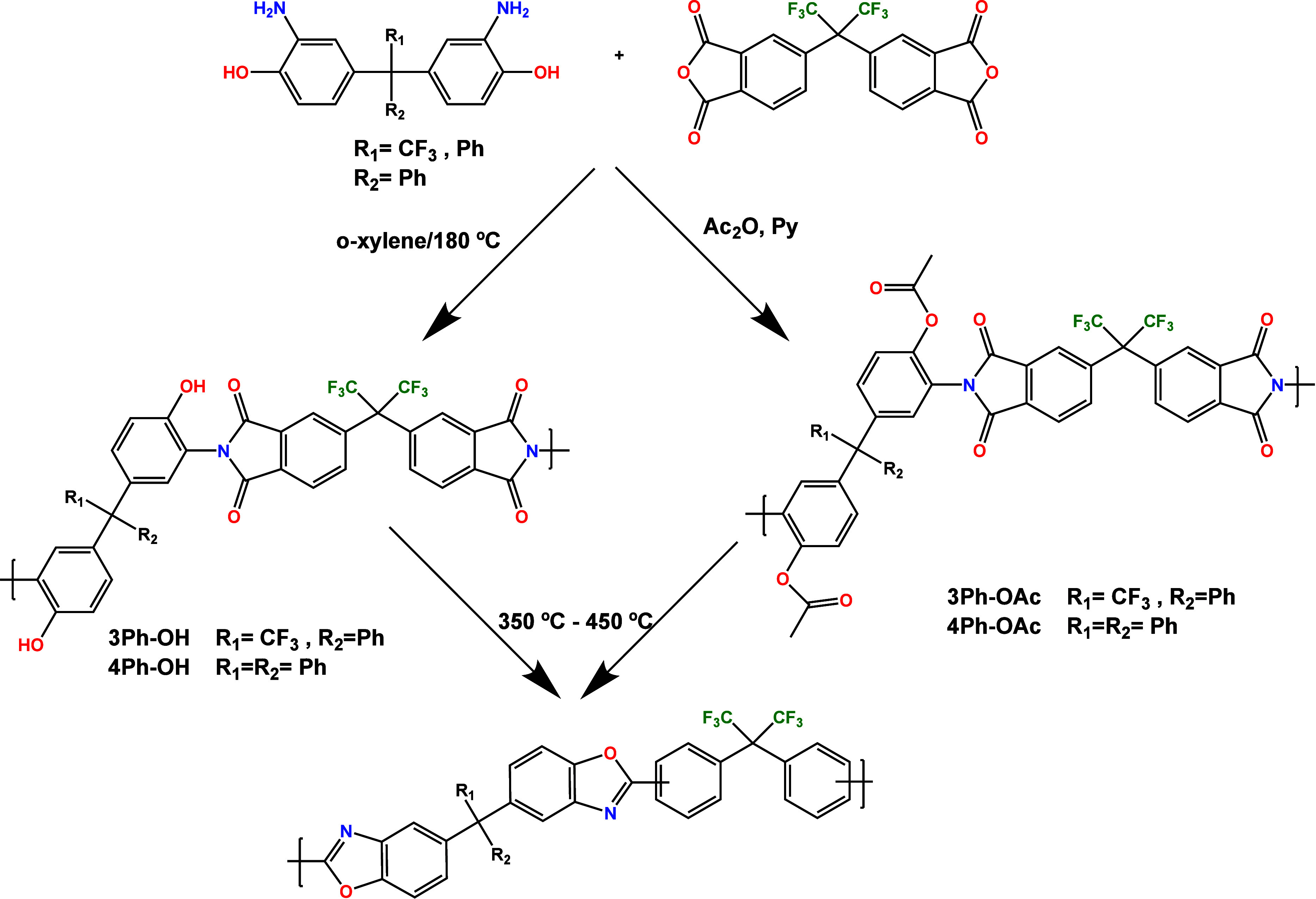
Scheme of the synthesis of precursor polyimides and their subsequent transformation to TR-PBOs.

The polyimides were prepared by a two-step, low-temperature polycondensation (Figure 3). The initial step consisted of the condensation reaction of diamine (**AH3P** or **AH4P**) with dianhydride (6FDA), where the diamines were subjected to an *in situ* silylation in order to increase their reactivities and obtain high molecular weight poly(amic acid) precursors.^25,34^ The details of the methodology, including the silylation agents and activators, are given in the Experimental Section. In the following step, the poly(amic acid)s were imidized by two different techniques, azeotropic and chemical cyclization. In the first one, an azeotropic agent (*o*-xylene) was used to remove the water and preserve hydroxyl groups, whereas the second one, where a mixture of acetic anhydride and Py was applied for cyclodehydration, resulted in polyimides with acetylated groups instead of OH. All of the precursors were obtained in quantitative yields and showed inherent viscosity values between 0.5 and 0.7 dL/g (Table 2), which were adequate for the preparation of self-supported dense membranes. The OH or OAc labels were added indicating the imidization route.

**Table 2:** Inherent Viscosity and Thermal Properties of the Precursor Polyimides

polymer	η_inh_ (dL/g)	*T*_g_ (°C)	theoretical weight loss (%)	measured weight loss^a^ (%)
3Ph-OH	0.71	264	11.3	13.2
3Ph-OAc	0.65	246	19.9	14.4
4Ph-OH	0.61	265	11.2	24.6
4Ph-OAc	0.52	255	19.7	18.8

a Determined by TGA at a heating rate of 10 °C/min under a nitrogen atmosphere.

**Figure 4:**
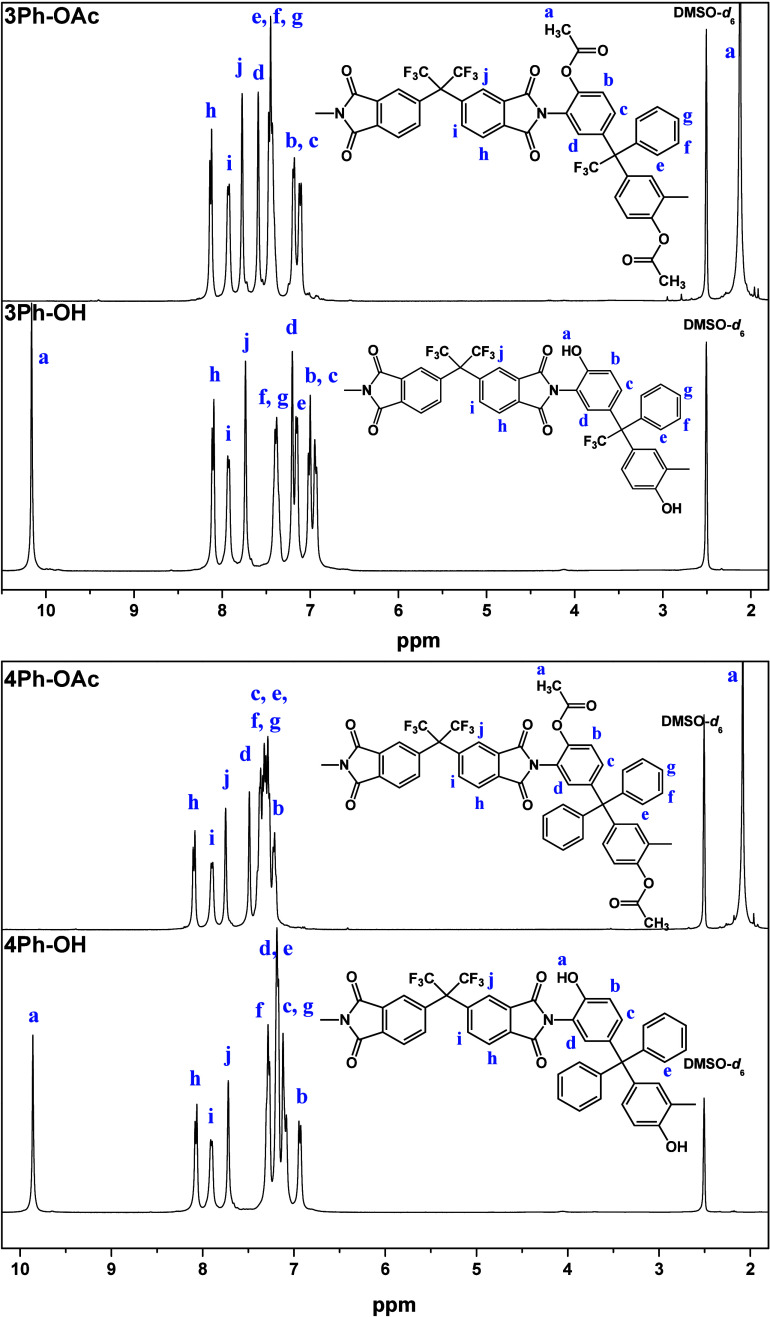
^1^H NMR spectra (400 MHz, DMSO-*d*_6_) of *o*-hydroxy and *o*-acetoxy polyimides.

Chemical structures of the *o*-hydroxy and *o*-acetoxy polyimides were confirmed by ^1^H NMR and FT-IR. Figure 4 depicts the ^1^H NMR spectra of the precursor polyimides. Both *o*-hydroxy polyimides present the OH signal above 9 ppm, whereas the proton from the acetate functionality appears around 2.08–2.12 ppm, and no signal from OH is detected for 3Ph-OAc and 4Ph-OAc, which confirms the complete acetylation of the hydroxyl groups. The aromatic proton resonances are placed between 6.8 and 8.2 ppm. No signals from the NH proton of the poly(amic acid) were detected, indicating the complete imidization of all the precursors. All the peak assignments are shown accordingly. The polyimides showed characteristic absorption bands in the FTIR spectra (Figure 8): the asymmetric and symmetric stretching vibrations of C=O (1780 and 1720 cm^–1^), the C–N stretching (1370 cm^–1^), the C–N–C transverse stretching at 1102 cm^–1^, and C–N–C out-of-plane bending at 720 cm^–1^. The *o*-hydroxy polyimides exhibited a broad band around 3400 cm^–1^ due to OH; additionally, the C–F stretching of the hexafluoroisopropylidene moiety was represented by absorption peaks around 1250–1100 cm^–1^.

### Thermal Properties of the Precursor Polyimides

Table 2 shows the glass transition temperatures (*T*_g_) of the polyimides’ films determined by DSC. *T*_g_ was affected by the *ortho* substituent. The polymers with the hydroxyl group presented the highest *T*_g_’s, and it seems that the values did not depend on the substituent in the diamine moiety, if it is phenyl or −CF_3_. However, as discussed below, residual solvent (THF), which was not possible to remove, may affect the *T*_g_’s values. The high *T*_g_’s of *o*-OH PIs are related to the ability of the hydroxyl groups to form hydrogen bonds and, thus, promote tight chain packing. The bulkiness of the acetoxy groups hindered the chain packing, which resulted in lower *T*_g_’s of *o*-OAc PIs. Besides, in contrast to *o*-OH PIs, *T*_g_ values of the *o*-OAc PIs were more sensitive to the polymer structure, and lower *T*_g_ = 246 °C was detected for the polyimide having a −CF_3_ group in the diamine fragment, 3Ph-OAc. For 4Ph-OAc with 4 phenyl rings in the diamine part, *T*_g_ was 10 °C higher (255 °C). It looks like the addition of a fourth phenyl substituent restricts the mobility of the chain to a greater extent when compared with the diamine having the CF_3_ group.

**Figure 5:**
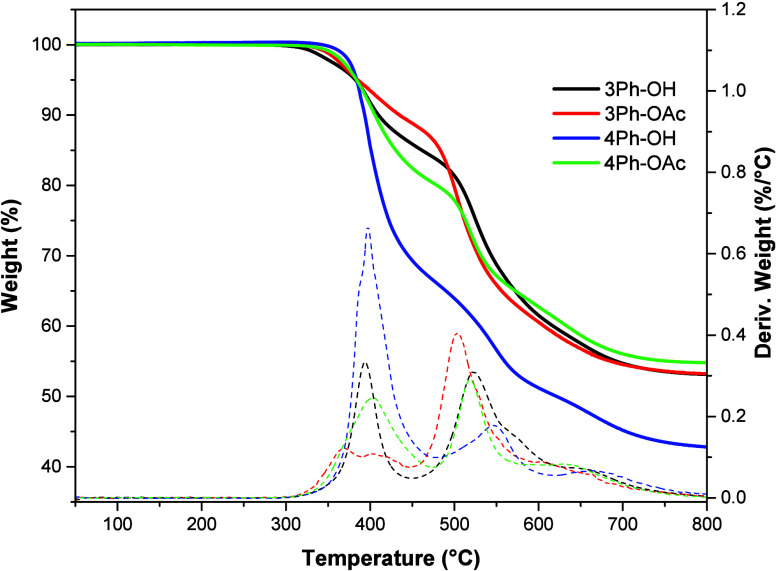
TGA and DTG curves of the precursor polymers.

Figure 5 displays TGA and differential thermogravimetric, DTG, curves for the precursor films at a heating rate of 10 °C/min under a nitrogen atmosphere. For all four polyimides, the TGA traces show two distinctive weight loss zones, consistent with the previous studies.^44^ The first stage, in the range 300–450 °C, is commonly associated with the thermal rearrangement of the polyimide precursor to polybenzoxazole accompanied by the release of CO_2_ molecules. The second stage starting between 450 and 480 °C presents the onset of the generalized thermal degradation of the polymer backbone. Three polymers, 3Ph-OH, 4Ph-OH, and 4Ph-OAc, have similar thermal rearrangement temperatures with a maximum weight loss of around 400 °C. Thus, the temperature at the maximum weight loss rate (*r*_WL_) for 3Ph-OH is 394 °C and *r*_WL_ = 0.31%/°C, for 4Ph-OH is 398 °C and *r*_WL_ = 0.53%/°C, and for 4Ph-OAc is 400 °C and *r*_WL_ = 0.24%/°C. For 3Ph-OAc this temperature is notably lower, 370 °C, with *r*_WL_ = 0.12%/°C. As has been shown, the conversion rate is strongly dependent on the precursor rigidity and the chain mobility,^39,45^ which generally agrees with the higher thermal rearrangement temperatures displayed by the studied polyimides having higher *T*_g_’s. In Table 2 one can observe the weight loss of each precursor obtained from TGA at 450 °C. The 4Ph-OH exhibited 24.6% weight loss, which is significantly above the theoretical value of 11.2% for the TR-PBO conversion, possibly due to residual solvent trapped within the polymer chains. However, the proximity between the rearrangement temperature and the temperature of solvent loss makes it difficult to remove completely. Moreover, the high *r*_WL_ = 0.53%/°C promotes the bubble formation on the film even at the lowest heat rate used, which made it impossible to obtain TR-PBO films of reasonable quality for this precursor at 425 and 450 °C. The measured weight losses for the other precursors, 3Ph-OH, 3Ph-OAc, and 4Ph-OAc, were 13.2, 14.4, and 18.8%, respectively, which were close to the theoretical values for the full conversion into TR-PBO as shown in Table 2.

**Figure 6:**
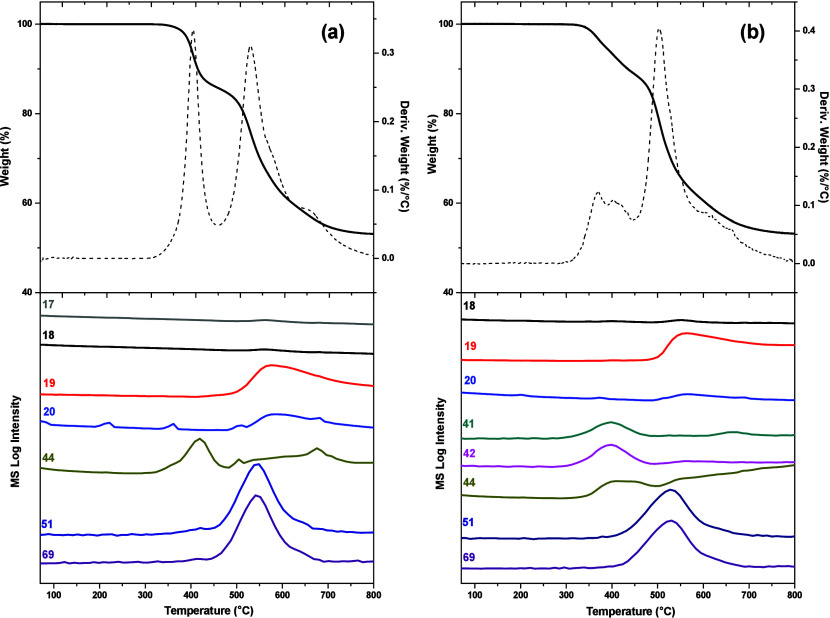
Thermogravimetric analysis combined with mass spectroscopy (TGA–MS) of (a) 3Ph-OH and (b) 3Ph-OAc precursor membranes (heating rate of 10 °C/min under a N_2_ atmosphere).

The compositions of the evolved gases during the rearrangement process were analyzed by TGA–MS, and the data are displayed in Figure 6 and Figures S3 and S4. During the thermal rearrangement (350–450 °C) of the *o*-hydroxy polyimides, CO_2_ is released as a product of the decarboxylation of the precursor, and this can be corroborated with the appearance of a single signal at 44 amu in the TGA–MS. Above 450 °C the degradation of the polymer backbone starts and MS species with 19, 20, and 69 amu appeared, indicating the cleavage of −CF_3_ groups, releasing F, HF, and CF_3_. In addition, peaks at 44 and 51 amu evolve, representing the degradation of the whole polyimide. The *o*-acetoxy polyimides exhibit similar behavior, except for the appearance of two signals during the rearrangement process with molecular weights of 41 and 42 amu related to the loss of the acetate groups, which are converted to ketene as the process reached high temperature.^24,46,47^ Since the experimentally found TR conversion for 4Ph-OH polymer did not coincide with the theoretical values (Table 2) and, additionally, very extensive bubble formation during heat treatment was observed, we supposed that it was due to residual solvent kept in the polymer matrix even after the pretreatment at 300 °C. Performing a more detailed TGA–MS study of this polymer (Figure S5), it was noted that during the first weight loss around (350–450 °C), other gases evolved besides the ones related to the formation of PBO. These MS species corresponded to 42, 28, and 16 amu, but the peak at 42 amu is the base peak in the THF mass spectrum. Moreover, the values of 28 and 16 amu could be related to ethylene and methane, which were reported as the principal products of the THF decomposition.^48−50^ We cannot conclude the exact reason why THF interacts very strongly with this particular polymer, whether it is due to CTCs, hydrogen bonds, or some other interactions.

### Thermal Treatment

**Figure 7:**
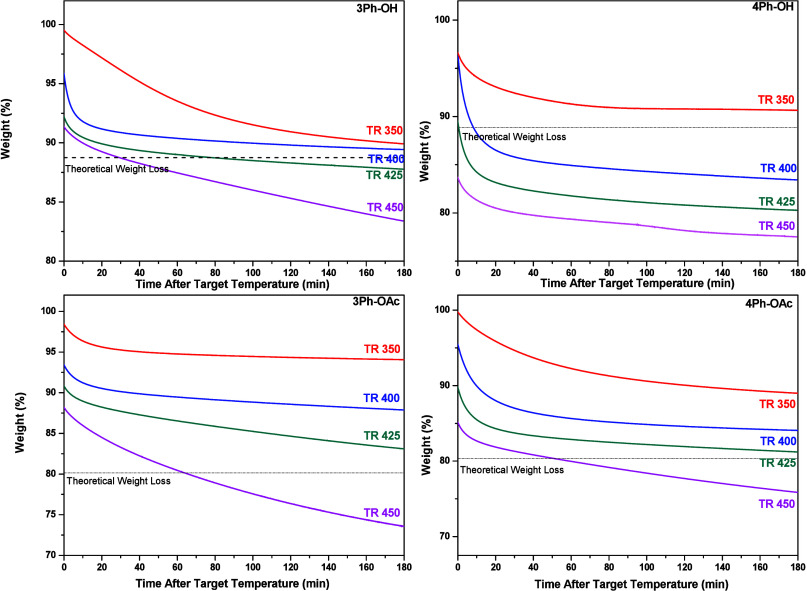
Isothermal thermogravimetric analysis of *o*-OH/*o*-OAc PIs membranes under a nitrogen atmosphere.

A more detailed TGA study was required to adjust the variables for the thermal treatment in the tubular furnace, and an isothermal study was performed. All the films were heated to 300 °C with a 5 °C/min rate and held at this temperature for 60 min with the intention to remove all residual solvent; then the films were heated further to the desired temperature (350 °C, 400 °C, 425 °C, and 450 °C) using the same rate of 5 °C/min and kept the films at that temperature for another 180 min. Figure 7 presents the isothermal thermograms of the precursors, showing the weight loss as a function of time and theoretical weight loss to the complete formation of TR-PBOs as dashed lines. The figure shows that the weight loss increases with an increase in the rearrangement temperature. For *o*-hydroxy polyimides, the weight loss is significantly higher than that for their *o*-OAc substituted analogs. At 350 °C, the weight loss for the OH precursors almost crossed the theoretical value. This temperature is not high enough to initiate a degradation process or a complete rearrangement; therefore, the weight lost is probably related to residual solvent trapped between polymer chains. However, for 4Ph-OH, the amount of solvent trapped was so elevated that its evaporation using any temperature regime prevented the obtention of quality membranes for thermal treatment temperatures above 400 °C. Several thermal treatment regimes were tested to avoid the formation of bubbles, but even using a very slow heating rate of 0.5 °C/min, the polymer film was damaged by bubble formation. For the *o*-OAc PIs, all the isotherms remained above the theoretical values except the ones at 450 °C. In comparison, the weight loss for 3Ph-OH precursor at 400 °C was very close to the theoretical value and crossed the line of whole conversion at 425 °C. Therefore, evaporation of the residual solvent from the OH precursor before the TRs temperatures were complicated, probably due to its more rigid structure, which reflects higher *T*_g_’s for the *o*-OH-PIs. At 450 °C, all of the samples present higher weight loss, exceeding the theoretical weight loss values at long treatment times. This means that thermal degradation and rearrangement processes may occur simultaneously. Based on these data and in order to have thermal histories similar to those reported in other TR studies, we selected the following thermal treatment regimes: 350 °C, 400 °C, and 425 °C for 1 h and 450 °C for 30 min. The percent conversion of the precursor’s membranes to TR-PBO was calculated from the isothermal study according to eq 6:
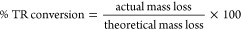
6

**Figure 8:**
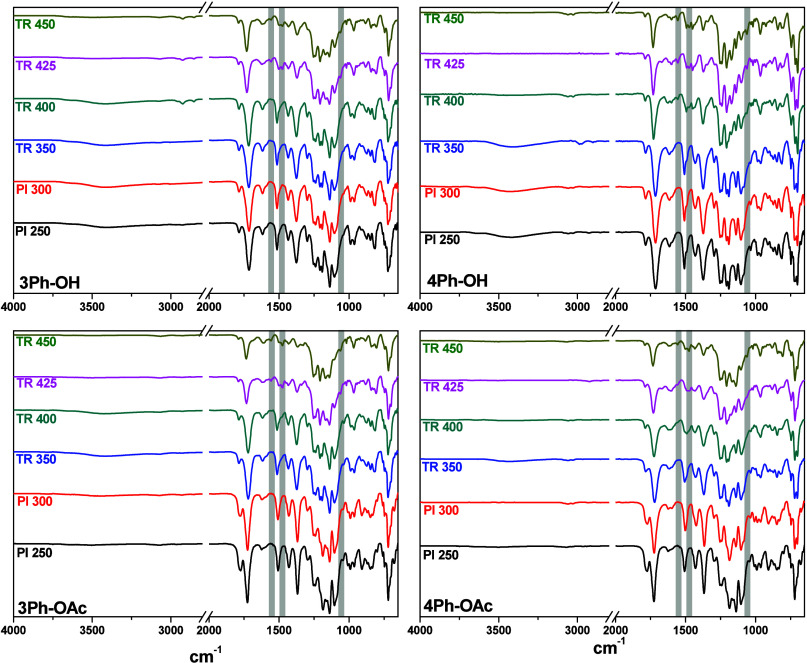
ATR-FTIR spectra of the precursors and their evolutions to TR-PBOs at different temperatures.

Table 3 displays the TR conversion values for the precursor polyimides, assuming that all weight loss was due to the thermal rearrangement. All the precursors pretreated at 300 °C did not show any notable TR conversion; at 350 °C TR conversions for *o*-OH precursors were higher than those for the *o*-OAc polyimides in agreement with their *r*_WL_. TR-conversions calculated for the 3Ph-OH and 4Ph-OH films were quite elevated at these temperatures of 58 and 78%, correspondingly; however, their FT-IR spectra (Figure 8) still showed the imide characteristic signals almost without changes. This agrees with our previous statement that the solvent was trapped strongly within *o*-OH substituted precursors and the weight loss observed was partially attributed to the evaporation of the residual solvent, particularly in the case of 4Ph-OH. At 400 °C, 3Ph-OH presented a TR conversion of 85% and total TR conversion at 425 and 450 °C. The conversion for 4Ph-OH was above the theoretical value; moreover, the rapid weight loss caused a detriment to the mechanical properties due to the extensive formation of bubbles during the thermal treatment. In the case of *o*-OAc polyimides, the evolution of TR conversion was much smoother, reaching high conversions at temperatures above 425 °C.

**Table 3:** Physical Properties of Precursor Polyimides and Their Corresponding TR Membranes

polymer code	% TR conversion	density (g·cm^-3^)	Vw (cm^3^·g^-1^)	FFV	*d*-spacing (nm)
3Ph-OH PI 250 °C	0	1.477	0.428	0.178	0.57
3Ph-OH PI 300 °C	0	1.479	0.428	0.178	0.58
3Ph-OH TR 350 °C	58	1.448	0.438	0.176	0.59
3Ph-OH TR 400 °C	85	1.425	0.443	0.180	0.59
3Ph-OH TR 425 °C	98	1.416	0.445	0.180	0.63
3Ph-OH TR 450 °C	100	1.394	0.446	0.193	0.63
4Ph-OH PI 250 °C	0	1.404	0.458	0.163	0.60
4Ph-OH PI 300 °C	0	1.409	0.458	0.160	0.61
4Ph-OH TR 350 °C	78	1.343	0.475	0.170	0.60
4Ph-OH TR 400 °C	>100	1.305	0.480	0.186	0.63
4Ph-OH TR 425 °C					
4Ph-OH TR 450 °C					
3Ph-OAc PI 250 °C	0	1.437	0.436	0.186	0.58
3Ph-OAc PI 300 °C	0	1.435	0.436	0.186	0.58
3Ph-OAc TR 350 °C	26	1.419	0.438	0.191	0.60
3Ph-OAc TR 400 °C	53	1.406	0.441	0.194	0.59
3Ph-OAc TR 425 °C	68	1.384	0.442	0.204	0.61
3Ph-OAc TR 450 °C	84	1.369	0.444	0.210	0.62
4Ph-OAc PI 250 °C	0	1.374	0.464	0.172	0.59
4Ph-OAc PI 300 °C	0	1.375	0.464	0.171	0.60
4Ph-OAc TR 350 °C	39	1.343	0.470	0.179	0.60
4Ph-OAc TR 400 °C	73	1.322	0.476	0.183	0.61
4Ph-OAc TR 425 °C	87	1.304	0.478	0.190	0.62
4Ph-OAc TR 450 °C	95	1.286	0.479	0.199	0.62

### Characterization of Thermally Rearranged Polybenzoxazole (TR-PBO) Membranes

The thermal rearrangement evolutions in the polymer films were followed by ATR-FTIR (spectra are displayed in Figure 8). All of the samples present a reduction in the intensity of the imide bands according to the rearrangement temperature, which can be associated with the TR conversion observed in TGA and isotherms. The *o*-OH precursor’s films showed no changes between 300 and 350 °C, however at 400 °C, small new peaks appeared around 1560 (C=N stretching, oxazole I), 1475, and 1060 (−O–C stretching) cm^–1^, consistent to the PBO structure, additionally, the broad absorption band corresponding to the OH at 3400 cm^–1^ started to decrease. The spectra of the *o*-OH films with thermal treatment above 400 °C have a more pronounced presence of the characteristic PBO bands and a gradual decrease in the OH peak intensity until its almost disappearance is also observed. The spectra corresponding to the *o*-OAc precursor films at 350 °C started to show a signal at 3400 cm^–1^ indicating that some of the acetoxy groups possibly convert to hydroxyl groups under the heating. Regarding their rearrangement into PBO structures, their behavior was similar to the *o*-OH PIs, although the characteristic PBO bands were less defined. There is a general agreement that *o*-hydroxy and *o*-acetoxy polyimides produce PBOs after thermal treatment; however, the mechanism of such transformation, particularly for *o*-acetoxy polyimides, is not clear, and some doubts about the structure of TR-polymers still exist. Thus, an alternate route was proposed suggesting the formation of rigid aromatic lactam units together with the formation of polybenzoxazoles when *o*-hydroxy polyimides were heated at temperatures above 400 °C.^51^ In this study, some samples treated at 450 °C presented a small band at 1675 cm^–1^ that could be associated with amide I of lactam, but the intensity was much lower than those related to PBO.

**Figure 9:**
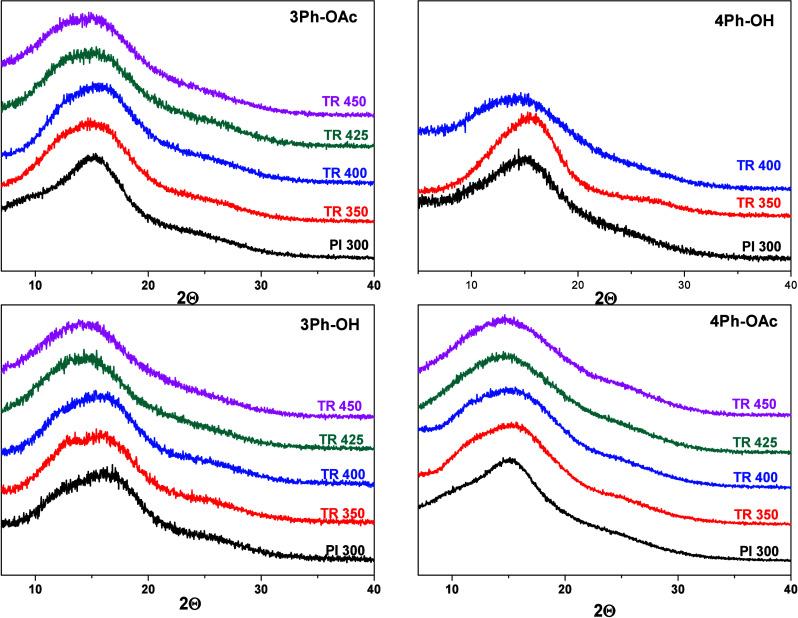
Wide-angle X-ray diffraction (WAXD) patterns of the precursors and TR-PBOs obtained at different temperatures.

Wide-angle X-ray diffraction (WAXD) was used to study the effect of thermal treatment on the chain packing of the precursors and TR-polymer films. Figure 9 shows the scattering patterns measured at room temperature. Broad featureless peaks or halos observed in the WAXD patterns of the samples reflected the amorphous structure of the polymers. Table 3 displays the values of preferential intersegmental distances (*d*-spacing) calculated with Bragg’s equation (eq 1). All pristine polyimides showed similar *d*-spacings, with values of 0.57–0.60 nm. As the temperature of thermal treatment increased, the amorphous halo of all the films became broader and the maximum was displaced slightly to lower angles, indicating an increase in the average intersegmental distances as a result of the formation of a more rigid benzoxazole backbone that inhibited the chain packing.

The density data of the precursors and TR membranes are shown in Table 3. The *o*-OH PIs present higher density values than their *o*-OAc analogs due to their ability to form hydrogen bonds. Likewise, as expected, a decrease in density is observed as the rearrangement temperature increases because of the formation of microcavities during the conversion to TR-PBO. Fractional free volumes (FFV) of the polymers were determined based on their density according to eq 3 are also shown in Table 3. The van der Waals volumes for partially rearranged samples were calculated considering the degree of conversion of the precursor to the final PBO structure using eq 7:
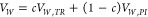
7where *c* is the fractional mass conversion determined by TGA with the help of eq 6; *V*_*W,TR*_ and *V*_*W,PI*_ are the van der Waals volumes of the TR-PBO and the polyimide precursor, respectively.

As can be seen from Table 3, for all the films, the FFV increases as the thermal rearrangement conversion grows, indicating a more open morphology after thermal treatment. The increment in FFV between the precursors and TR membranes treated at 450 °C was 8% for OH-substituted and 13–15% for *o*-OAc PIs. The higher FFV values for TR-PBOs derived from *o*-OAc PIs are rather related to the elimination of larger groups during thermal rearrangement.^26,44,46^

### Mechanical Properties

The mechanical properties of the membranes in this study are displayed in Table 4. All the precursors exhibited good mechanical properties with Young’s modulus higher than 2.4 GPa and tensile strength up to 100 MPa, aside from the 4Ph-OH, which had a large amount of residual solvent. The membranes treated at 300–350 °C practically did not change their mechanical properties in relation to the ones heated at 250 °C, except for 4Ph-OH, whose mechanical properties quickly decayed. The large amount of solvent trapped in the 4Ph-OH film evaporated very quickly during thermal rearrangement, causing bubbles and, as a consequence, a drastic reduction of the mechanical properties that made the measurement of this membrane impossible. As for the other membranes, the treatment at 400 °C produced only a small decrease in the mechanical properties, and their values remained in the range reported for other aromatic polyimides.^35−37^ However, the mechanical properties of the membranes treated at temperatures higher than 425 °C declined considerably, especially for OAc polymers; the elongation values were lower than 2.6% and the tensile strengths were reduced by approximately 80%. Although the rearrangement process decreased the mechanical properties, of the membranes, uniform self-supported TR-membranes were obtained, and it was possible to measure their gas separation properties, except for 4Ph-OH.

**Table 4:** Mechanical Properties of the Precursors and TR Membranes

polymer	Young’s modulus (GPa)	elongation at break (%)	tensile strength (MPa)
3Ph-OH PI 250 °C	3.3	12.0	108.1
3Ph-OH PI 300 °C	2.9	11.8	106.3
3Ph-OH TR 350 °C	2.2	9.1	102.0
3Ph-OH TR 400 °C	2.1	8.7	83.7
3Ph-OH TR 425 °C	2.2	3.2	53.3
3Ph-OH TR 450 °C	1.9	2.6	46.8
4Ph-OH PI 250 °C	2.4	5.2	81.7
4Ph-OH PI 300 °C	2.3	4.7	73.3
4Ph-OH TR 350 °C	0.9	0.8	28.8
4Ph-OH TR 400 °C	0.6	0.5	7.7
3Ph-OAc PI 250 °C	3.5	10.1	132.0
3Ph-OAc PI 300 °C	3.3	8.3	111.6
3Ph-OAc TR 350 °C	3.1	8.1	102.8
3Ph-OAc TR 400 °C	2.7	7.0	79.8
3Ph-OAc TR 425 °C	2.3	2.3	36.2
3Ph-OAc TR 450 °C	1.5	1.2	27.8
4Ph-OAc PI 250 °C	4.5	8.0	102.4
4Ph-OAc PI 300 °C	4.0	7.4	91.7
4Ph-OAc TR 350 °C	3.3	6.7	77.2
4Ph-OAc TR 400 °C	2.7	5.9	65.2
4Ph-OAc TR 425 °C	3.0	1.7	40.4
4Ph-OAc TR 450 °C	1.8	1.3	29.0

### Gas Permeation Properties

**Figure 10:**
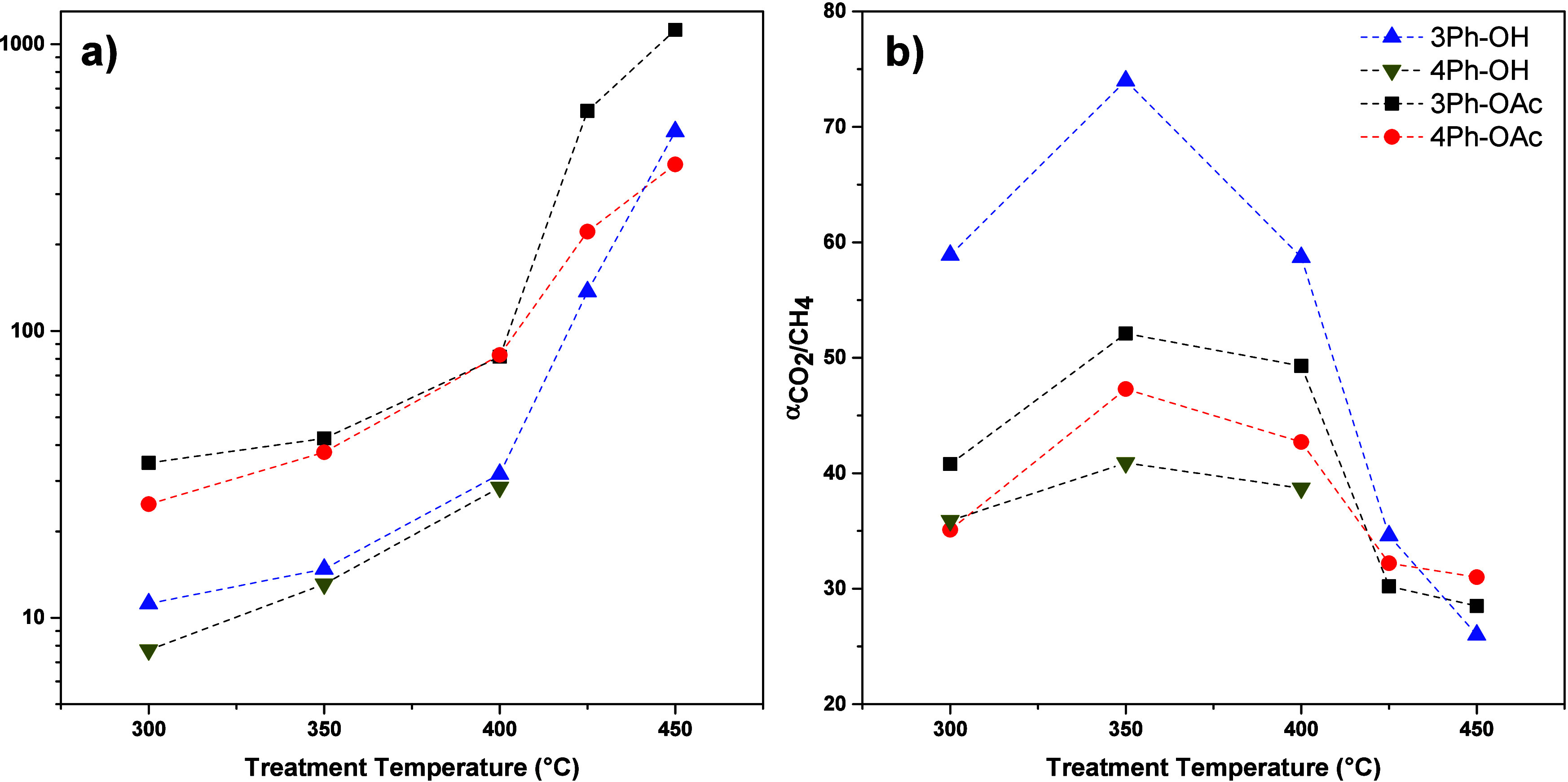
Dependence of the CO_2_ permeability (a) and CO_2_/CH_4_ selectivity (b) of the membranes on the treatment temperature.

Pure gas permeability and selectivity coefficients for the precursors and TR membranes measured at 3 bar and 35 °C are given in Table 5. The precursor polyimides have permeability values similar to other polyimides reported;^34,46^ however, it can be seen for our polymers that the type of *ortho*-substituent influences the permeability; the *o*-OAc PIs have a slightly higher permeability than *o*-OH PIs. This agrees with the difference in FFV between them since the relatively bulky acetoxy groups generate a greater disruption in the polymer packaging than the OH groups. As for the TR-PBO membranes, the permeability for all the gases increases as the rearrangement temperature increases independently of the precursor. This effect is shown graphically for the CO_2_ permeability in Figure 10a for better visualization. Regarding ideal selectivity, the values for CO_2_/CH_4_ (Figure 10b) increased at 350 °C, because the chains had more mobility at this temperature, causing a redistribution of the FFV and therefore an increase in selectivity. However, the selectivities for CO_2_/CH_4_ dropped as the treatment temperature increased beyond 400 °C, which coincided with the increase in the FFV in TR-PBOs. Since the thermal rearrangement process of hydroxy- and acetoxypolyimides is different and carried out at different temperatures, it is convenient to investigate how the permeability changes according not only to the thermal treatment temperature but also to the TR conversion. Figure S6 shows the increase in the CO_2_ permeability as a function of TR conversion for the four polymers studied. For 3Ph-OH the increase in permeability was quite low and a large increase in permeability was achieved only at high TR conversions, with a maximum increment of 40 times at almost 100% TR conversion. On the other hand, the 3Ph-OAc polymer presented a different behavior, with an increase in permeability from lower conversion (65%) and a 32-fold increment at TR conversion of 85%. This difference between the *o*-OAc and *o*-OH polymers may be explained by the differences in the rearrangement processes and the loss of bulkier side groups. According to TGA–MS and FT-IR analyses (Figures 6 and 8), the loss of the side groups in the *o*-OAc PIs were observed in the form of acetyls or ketals before starting the main thermal rearrangement. Simultaneously, the appearance of OH functionalities was detected in these polymers, which may also participate in the TR-PBO rearrangement. Therefore, an increase in FFV occurs without necessarily having a high TR conversion.

**Table 5:** Pure Gas Permeation Properties of Precursors and TR membranes, Measured at 35 °C and 3 bar upstream Pressure

	permeability (barrer)^a^					ideal selectivity	
polymer	He	O_2_	N_2_	CH_4_	CO_2_	α_O2/N2_	α_CO2/CH4_
3Ph-OH PI 300 °C	67	3.3	0.56	0.19	11	5.9	59
3Ph-OH TR 350 °C	77	3.8	0.61	0.20	15	6.3	74
3Ph-OH TR 400 °C	116	5.9	0.94	0.54	32	6.3	59
3Ph-OH TR 425 °C	218	31	6.4	4.0	137	4.8	35
3Ph-OH TR 450 °C	372	99	25	19	496	3.9	26
4Ph-OH PI 300 °C	71	3.9	0.68	0.22	7.7	5.8	36
4Ph-OH TR 350 °C	89	5.3	0.91	0.32	13	5.8	41
4Ph-OH TR 400 °C	125	12.5	2.3	0.75	29	5.4	39
3Ph-OAc PI 300 °C	97	8.3	1.6	0.85	35	5.3	41
3Ph-OAc TR 350 °C	134	10.7	1.9	0.81	42	5.7	52
3Ph-OAc TR 400 °C	177	19.0	3.5	1.7	81	5.4	49
3Ph-OAc TR 425 °C	468	123	31	19	585	4.0	30
3Ph-OAc TR 450 °C	730	270	71	39	1121	3.8	29
4Ph-OAc PI 300 °C	81	6.8	1.2	0.71	25	5.5	35
4Ph-OAc TR 350 °C	105	8.7	1.5	0.80	38	5.7	47
4Ph-OAc TR 400 °C	142	22	4.2	1.9	83	5.2	43
4Ph-OAc TR 425 °C	225	38	7.4	6.9	222	5.1	32
4Ph-OAc TR 450 °C	396	81	18	12	381	4.6	31

a 1 barrer = 10^–10^ cm^3^ (STP) cm cm^–2^ s^–1^ cmHg^–1^.

**Figure 11:**
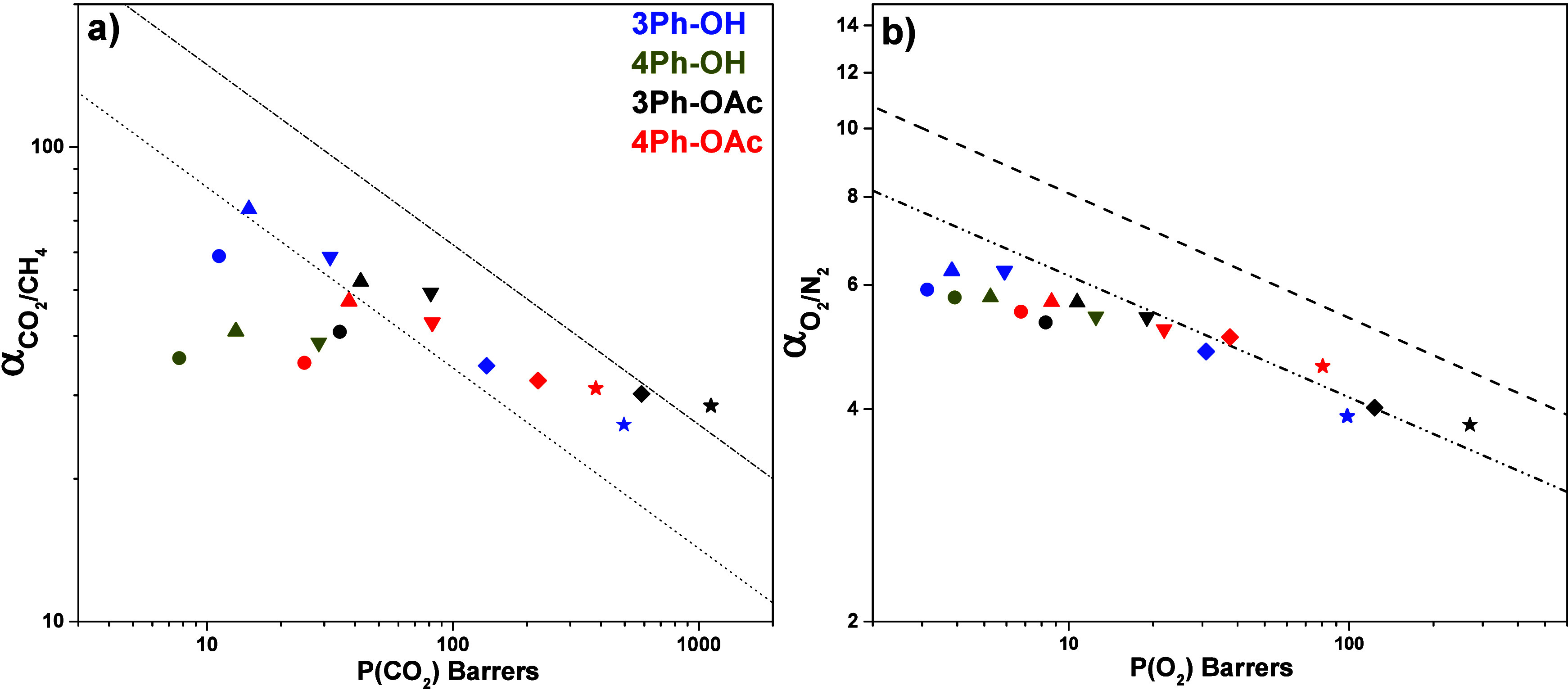
Separation performance for polyimide precursors and TR-PBO’s membranes for (a) CO_2_/CH_4_ and (b) O_2_/N_2_; ● = 300 °C, ▲= 350 °C, ▼= 400 °C, ◆ = 425 °C and ★ = 450 °C. 1991 and 2008 Robeson upper bond plots are shown as dotted-dash and dashed lines, correspondingly.

The CO_2_/CH_4_ and O_2_/N_2_ separation performance of the studied membranes is shown in Figure 11. For CO_2_/CH_4_, thermal rearrangement at 350 °C produced membranes with high selectivity that exceeded the 1991 upper bound.^52^ Further increase in the temperature led to an additional increment in permeability but a decrease in selectivity; however, as the temperature rises, the permselectivities are getting closer to the 2008 Robeson upperbound. 3Ph membranes with a pendant CF_3_ group exhibited better performance than their counterparts 4Ph bearing additional Ph substituent instead, which agrees with the calculated FFV values. In the same way, TR-PBO membranes from *o*-OAc PIs showed higher permeabilities than those from *o*-OH PIs. Such a combination makes 3Ph-OAc TR-450 °C film a membrane with the best performance, surpassing the Robeson 2008 upper bound.^53^ Regarding the O_2_/N_2_ gas pair, most of the membranes are placed below the 1991 Robeson upper bound. The effect of the thermal rearrangement for this pair of gases is less noticeable; nevertheless, *o*-OAc TR-450 °C membranes exceed the 1991 limit.

## Conclusions

Two diamines with bulky CF_3_ and phenyl groups were synthesized in high yields and purity. These monomers were used to obtain a series of *o*-hydroxy and *o*-acetoxy polyimides of high molecular weights based on 6FDA dianhydride. These polymers formed membranes with good thermal and mechanical properties. Thermal rearrangement of the membranes to polybenzoxazoles in the temperature range of 350–450 °C was studied using TGA–MS and ATR-FTIR analyses. Thermal rearrangement conversions increased with temperature and were found to be higher for *o*-OH substituted precursors. The fractional free volume, FFV, of the TR-PBO polymers increased simultaneously with TR conversion, and the growth was higher for the *o*-OAc substituted precursors. WAXD data also showed *d*-spacing growth, which agreed with the increase in FFV observed in the TR-PBO membranes. The mechanical properties of the TR-membranes only slightly diminished up to 400 °C concerning their precursors but significantly deteriorated at 450 °C. Despite this, it was possible to obtain TR-PBO membranes of good quality even after the 450 °C treatment, except for the 4Ph-OH precursor, where extensive bubble formation was observed due to significant evaporation of the solvent trapped within this polymer.

Finally, the precursors and TR-PBOs were evaluated for their pure gas separation properties. In all cases, the permeability of the measured gases augmented according to the increase in the rearrangement temperature. For CO_2_, up to a 40-fold increase was found for the TR-450 °C obtained from 3Ph-OH. Regarding selectivity, at temperatures higher than 400 °C, there was a decrease in the ideal selectivity for CO_2_/CH_4_. In the case of the TR-PBO obtained from 3Ph-OAc at 450 °C, the permeability vs permselectivity plot exceeded the upper bound established by Robeson in 2008.

The relatively simple synthesis of these polymers from inexpensive and accessible reagents and the good gas separation properties of these polymeric materials make them a potential option for large-scale industrial applications.

## Supporting Information Available

^13^C NMR spectra of AH3P and AH4P; TGA–MS of polyimides 4Ph-OH and 4Ph-OAc; TGA–MS for trapped solvent in 4Ph-OH polymer; and permeability of CO_2_ as a function of TR conversion for all the polymers (PDF)
