# Supplementary material for: Thermally Rearranged (TR) Polybenzoxazoles from o-Substituted Precursor Polyimides with Phenyl Pendant Groups: Synthesis, Properties, and Thermal Rearrangement Conditions
Source: Macromolecules. 2024 Aug 6;57(16):8187–201. doi: 10.1021/acs.macromol.4c00169 (PMC11363616; doi:10.1021/acs.macromol.4c00169)
Supplement: Supplementary file 1 — ma4c00169_si_001.pdf [file ma4c00169_si_001.pdf]

Supporting Information

Thermally Rearranged (TR) Polybenzoxazoles from *o*-substituted Precursor Polyimides with Phenyl Pendant Groups: Synthesis, Properties and Thermal Rearrangement Conditions.

Mario Rojas-Rodriguez[1], Sandra Rico-Martínez[2], Pedro Prádanos[3], Cristina Álvarez[4], Larissa Alexandrova[1], Young Moo Lee[5,*], Ángel E. Lozano[2,3,4,*], Carla Aguilar-Lugo[1,4*]

[1] Instituto de Investigaciones en Materiales, Universidad Nacional Autónoma de Mexico, Circuito Exterior S/N, Ciudad Universitaria, 04510, CDMX, Mexico

[2] Instituto Universitario CINQUIMA, University of Valladolid, Paseo Belén 5, 47011, Valladolid, Spain.

[3] SMAP, Associated Research Unit to CSIC, Faculty of Science, University of Valladolid, Paseo Belén 7, 47011 Valladolid, Spain

[4] Instituto de Ciencia y Tecnología de Polímeros, ICTP-CSIC, Juan de la Cierva 3, E-28006 Madrid, Spain.

[5] Department of Energy Engineering, College of Engineering, Hanyang University, Seoul 04763, Republic of Korea

*Corresponding authors:, Young M. Lee; email: ymlee@hanyang.ac.kr, Ángel E. Lozano; email: lozano@ictp.csic.es, and Carla Aguilar-Lugo; email: carla.aguilar@iim.unam.mx

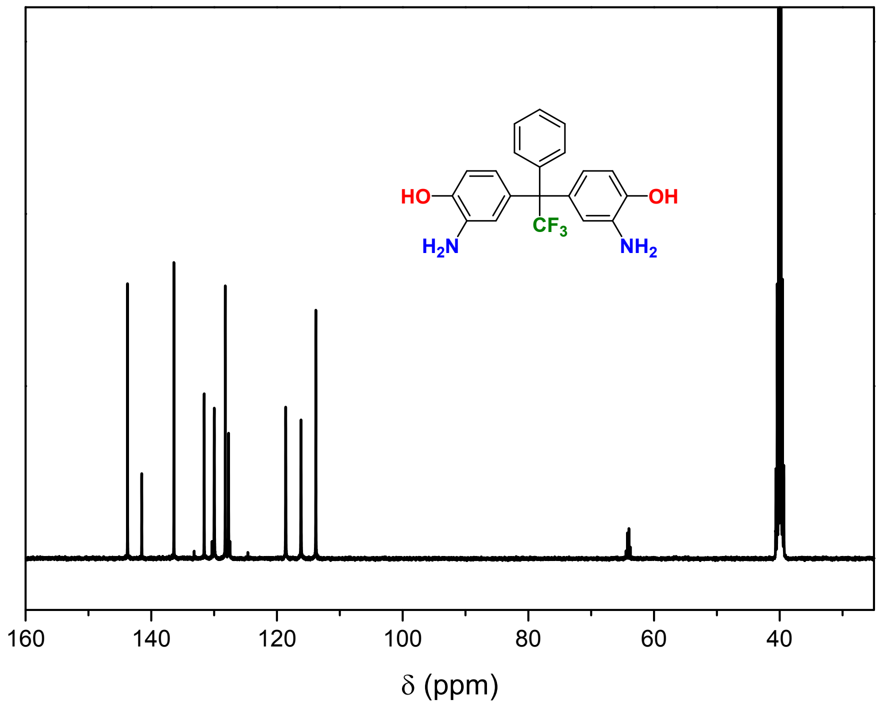

Figure S1. ^{13}C NMR spectrum (DMSO-d_6) of AH3P.

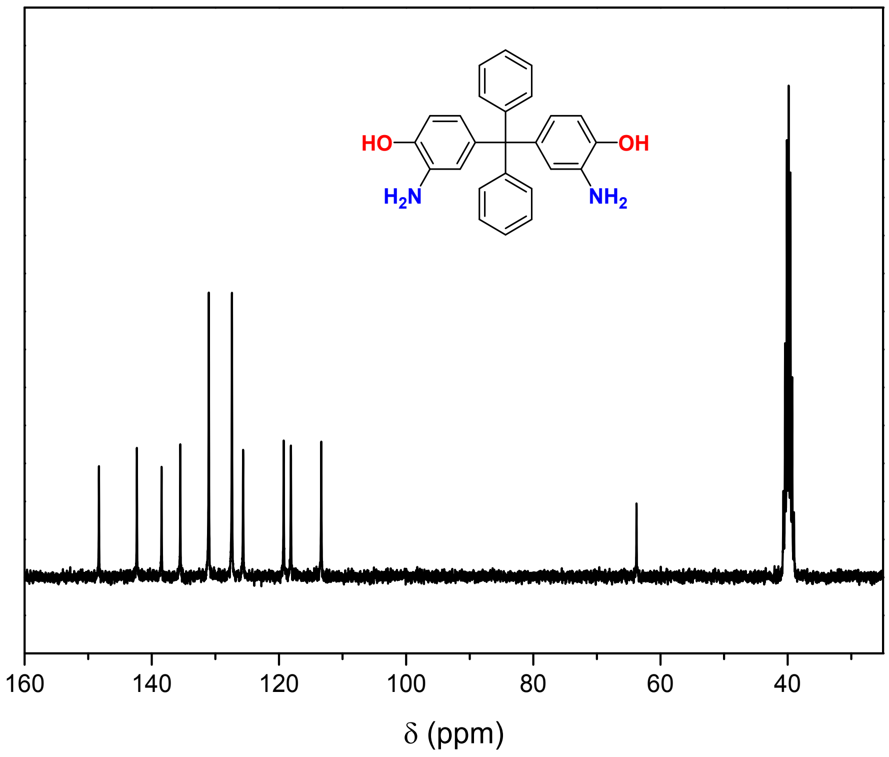

Figure S2. ^{13}C NMR spectrum (DMSO-d_6) of AH4P.

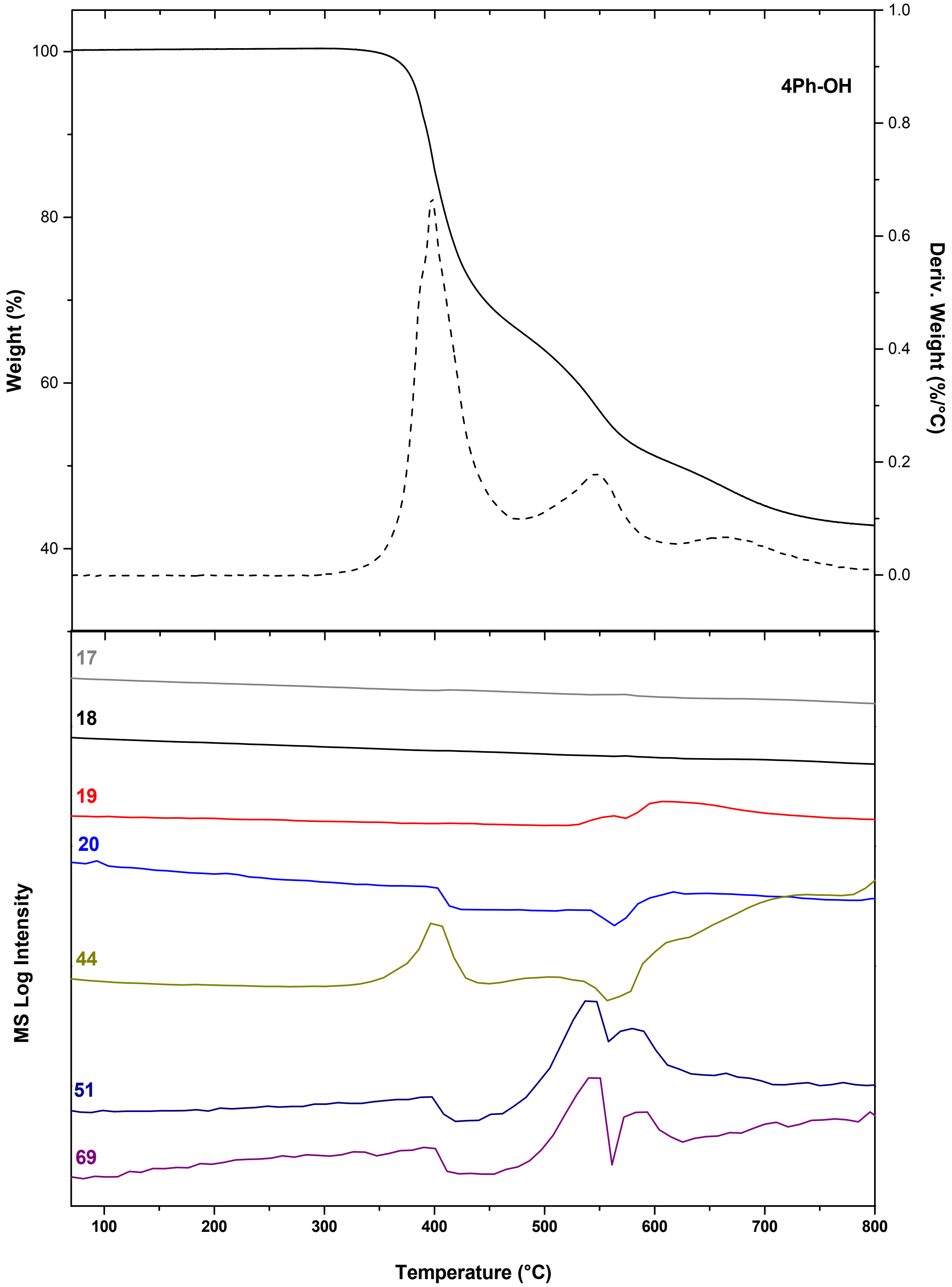

Figure S3. TGA-MS of 4Ph-OH polymer.

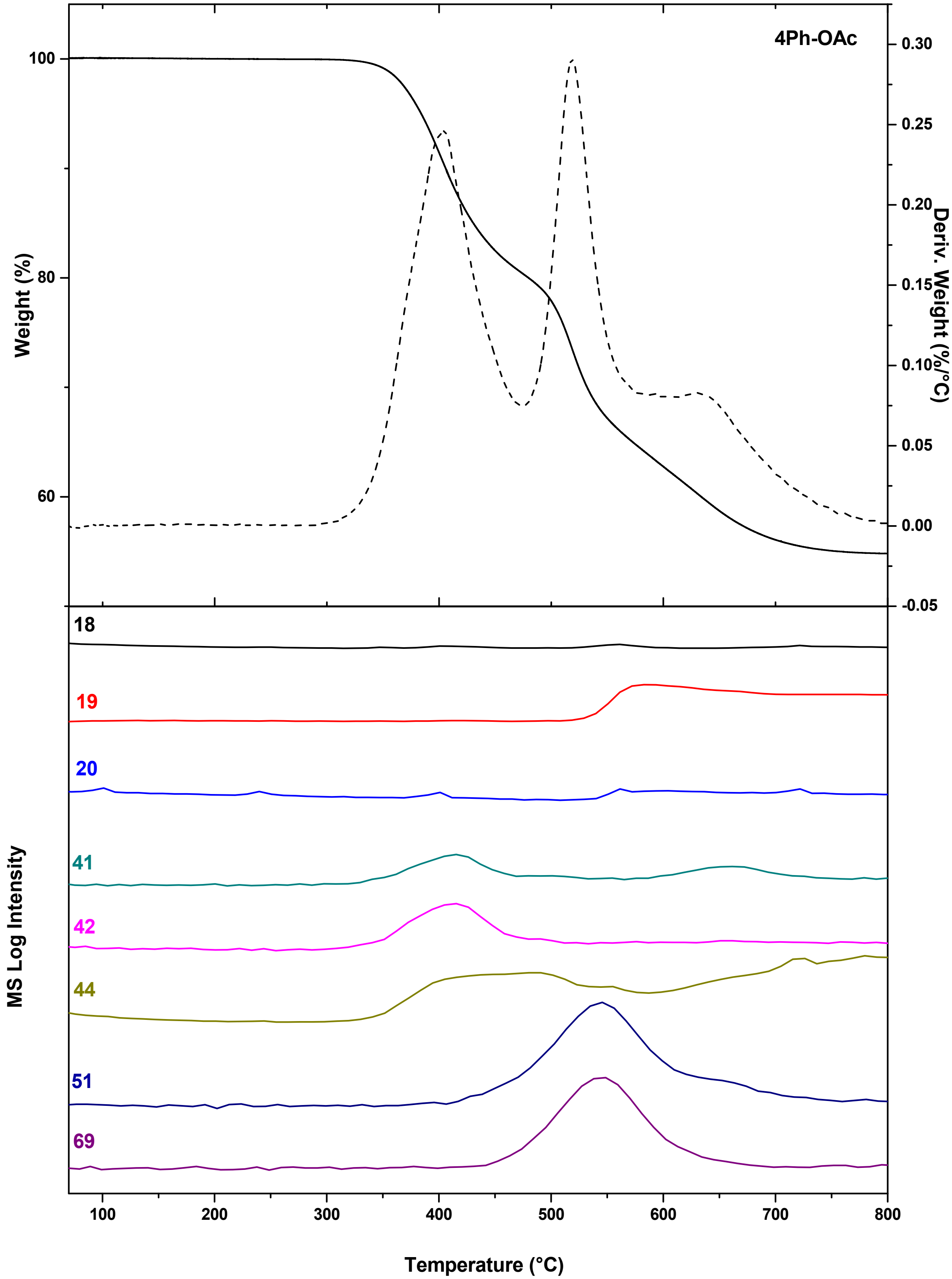

Figure S4. TGA-MS of 4Ph-OAc polymer.

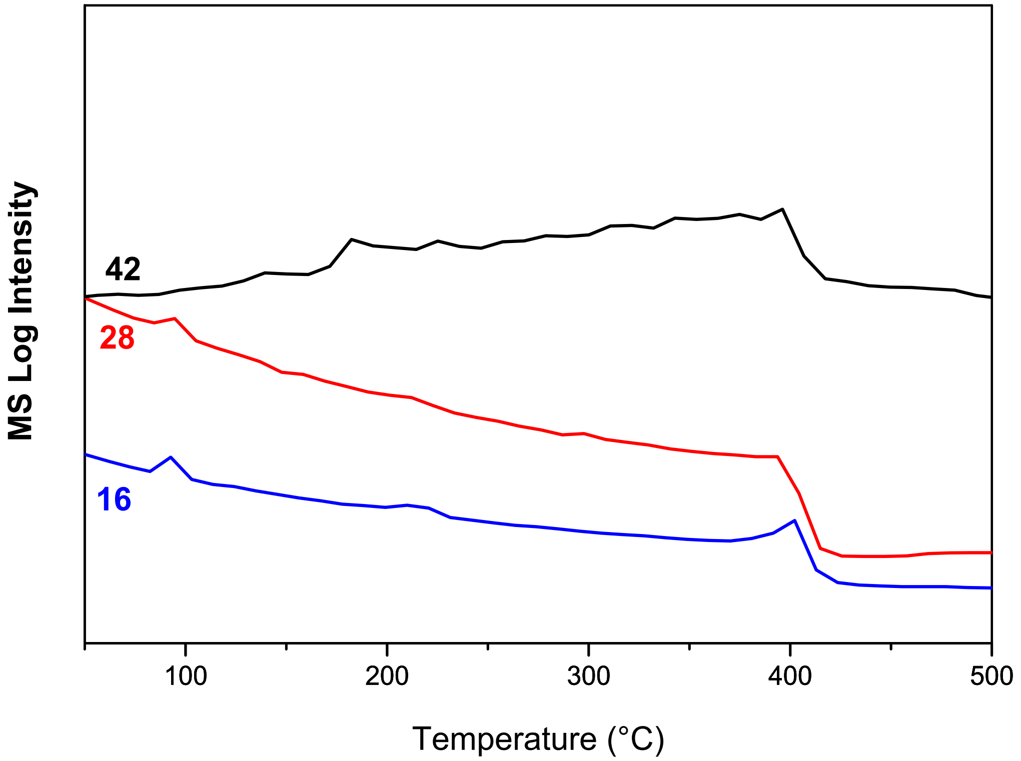

Figure S5. Mass Analysis for trapped solvent in 4Ph-OH.

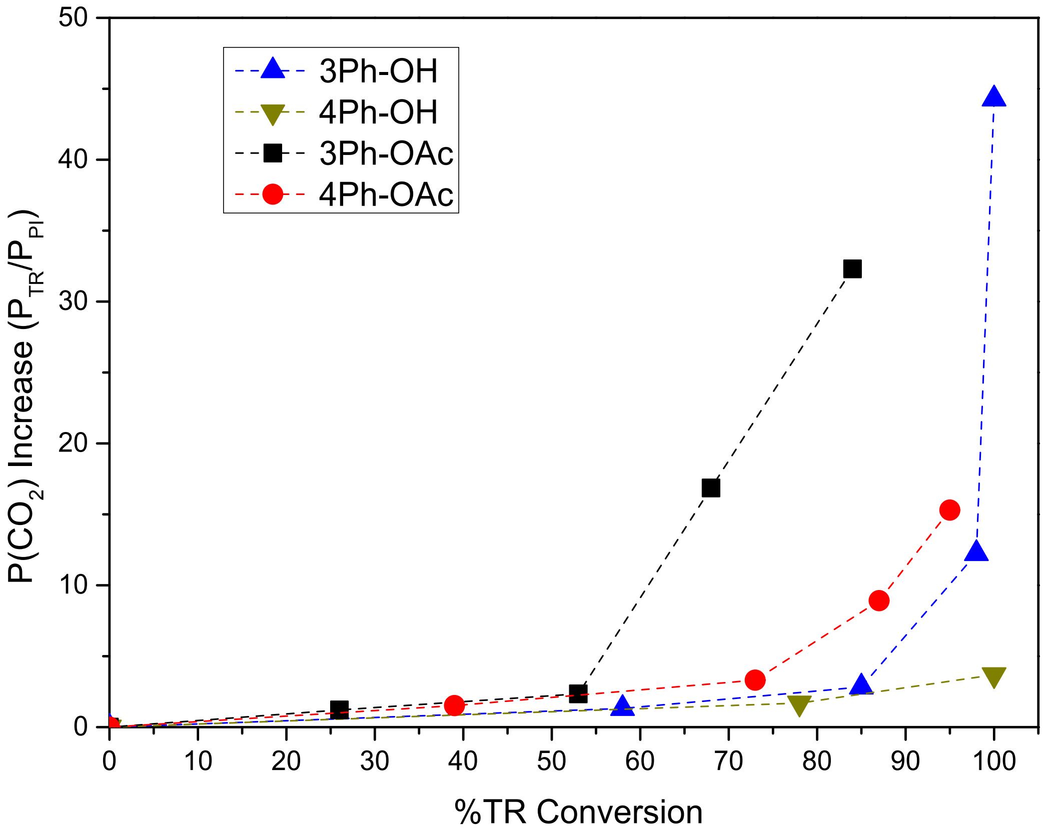

Figure S6. CO_2 permeability increment as a function of TR conversion.
